# Precession electron diffraction – a topical review

**DOI:** 10.1107/S2052252514022283

**Published:** 2015-01-01

**Authors:** Paul A. Midgley, Alexander S. Eggeman

**Affiliations:** a Department of Materials Science and Metallurgy, University of Cambridge, 27 Charles Babbage Road, Cambridge CB3 0FS, England

**Keywords:** precession electron diffraction (PED), electron crystallography, electron techniques, electron-based structure analysis

## Abstract

In the 20 years since precession electron diffraction (PED) was introduced, it has grown from a little-known niche technique to one that is seen as a cornerstone of electron crystallography. It is now used primarily in two ways. The first is to determine crystal structures, to identify lattice parameters and symmetry, and ultimately to solve the atomic structure *ab initio*. The second is, through connection with the microscope scanning system, to map the local orientation of the specimen to investigate crystal texture, rotation and strain at the nanometre scale. This topical review brings the reader up to date, highlighting recent successes using PED and providing some pointers to the future in terms of method development and how the technique can meet some of the needs of the X-ray crystallography community. Complementary electron techniques are also discussed, together with how a synergy of methods may provide the best approach to electron-based structure analysis.

## Abstract 2

This topical review highlights progress made recently in the development and application of precession electron diffraction (PED) and its scanning variant for the determination of unknown crystal structures and the mapping of orientations at the nanoscale.

## 1. Introduction   

A modern (scanning) transmission electron microscope, or (S)TEM, is a remarkably versatile and powerful instrument. With the advent of aberration correctors, monochromators and computer control, the electron microscope is able to form not only ultra-high resolution images but also two- and three-dimensional maps of composition [using electron energy-loss spectroscopy (EELS) and energy-dispersive X-ray spectrometry (EDS)] and of electromagnetic fields (through electron holography), and can follow time-resolved phenomena at the sub-picosecond timescale (Midgley & Thomas, 2014 ▶). Electron diffraction patterns are easily formed by making the final image plane conjugate with the back focal plane of the objective lens using post-specimen lenses. The strong Coulombic interaction between the electron beam (typically with energies 80–300 keV) and the electrostatic potential of the sample leads to diffraction intensities that are, in general, exquisitely sensitive to the underlying crystal symmetry and the thickness of the specimen. For typical samples and thicknesses studied, this strong interaction leads to a high probability of multiple (or dynamic) scattering and thus diffracted intensities that cannot, in general, be interpreted within a simple kinematic framework. For structure solution this is problematic, as all standard solution algorithms rely on the fact that the recorded intensities are proportional to the (square of the) structure factor for that reflection and, just as importantly, that the intensity of one reflection does not depend on the structure factor of other reflections.

For many years, using electron diffraction intensities to ‘solve’ crystal structures relied on being able first to guess, or to have prior knowledge of, a likely model structure and then to refine that structure (and often the sample thickness), taking dynamic effects into account, to best match computed diffracted intensities with experimental ones. Whilst this led to many successful refinements, including not only atomic positions (Vincent *et al.*, 1984 ▶) but also refinements of charge bonding densities (Midgley & Saunders, 1996 ▶; Zuo *et al.*, 1999 ▶), *ab initio* structure determination was problematic.

**Figure 1:**
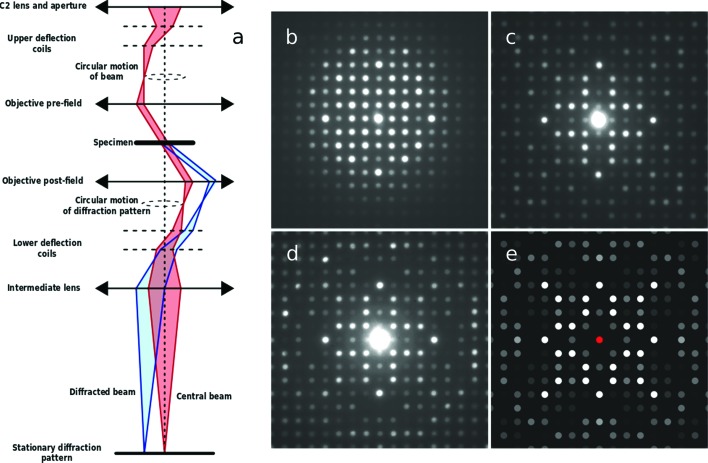
(*a*) A schematic ray diagram for precession electron diffraction (PED), illustrating the rocking/de-rocking action of the beam before and after the specimen. (*b*)–(*e*) Illustrations of how precession alters the recorded diffracted intensities, here from the [001] zone axis of Er_2_Ge_2_O_7_: (*b*) without precession, (*c*) with a precession angle of 20 mrad, (*d*) with a precession angle of 47 mrad. The pattern of diffracted intensities seen in (*d*) is similar to that seen in the kinematic simulation shown in part (*e*).

To tackle that problem, precession electron diffraction (PED) was introduced 20 years ago as a way of recording electron diffraction intensities more suited to structure solution than those acquired with conventional methods (Vincent & Midgley, 1994 ▶). As shown in Fig. 1 ▶, the electron beam is rocked in a hollow cone (at a fixed angle to the optic axis) above the sample and then de-rocked below the sample, the net effect being equivalent to precessing the sample about a fixed electron beam parallel to the optic axis. The resultant pattern is composed of many more reflections than would be the case for an unprecessed beam, and these PED reflections have intensities that are determined by integrating through the Bragg condition of each reflection. In early papers (Vincent & Midgley, 1994 ▶), the high-order Laue zone (HOLZ) reflections were used to determine (conditional) Patterson maps and then atomic positions, but it was soon realised that even the zero-order Laue zone (ZOLZ) reflections could be used (with care) to determine structures *ab initio* using conventional X-ray phasing methods and structure solution programs. At first this seems surprising, given the inherent dynamic effects in any electron diffraction pattern. However, as will be discussed further below, even though individual intensities are not kinematic, the ensemble of intensities in a PED pattern behaves in a ‘kinematic-like’ fashion, at least sufficiently so to enable successful structure solution.

The use of PED has grown within the electron microscopy community and, importantly, is now seen as a method that complements those of X-ray diffraction, especially when examining multi-phase samples, disordered crystals or other systems that, for whatever reason, may not be amenable to conventional X-ray crystallographic analysis.

In this topical review, we focus on developments in the understanding of why PED enables structure solution, on the best use of the technique and on its application across a wide range of specimens and structures. Since its inception 20 years ago, PED has become an invaluable tool, not only for the determination of unknown crystal structures but also in aiding the analysis of local microstructure, particularly through a combination of PED and beam scanning to map relative crystal orientation, texture, strain *etc*. The brevity and topical focus of the review preclude a detailed exposition of the history of PED and how it complements other electron diffraction methods, but an interested reader may wish to consult a longer paper published by the authors two years ago (Eggeman & Midgley, 2012*b* ▶).

## 2. Recent developments for structure solution   

Throughout the past ten years or so there has been a steady stream of papers using PED to solve structures that could not be solved through conventional X-ray (or neutron) methods. In parallel, there has been a great deal of work undertaken to try to establish the best conditions for PED, especially in terms of precession angle. However, almost uniformly across all the PED structure solutions has been the fact that, whilst the structures first solved, and then refined, are plausible, the *R* factors used to ascertain the quality of the structure have remained high (typically 0.20–0.30), far higher than most equivalent X-ray refinements.

Early PED work suggested that the use of ZOLZ reflections was nearly always going to lead to poor refinements because of inherent dynamic effects, prevalent in low-order reflections but less so in the HOLZ. More detailed analysis of the behaviour of ZOLZ reflections showed that, whilst individual reflections could deviate a great deal from their kinematic value, for sufficiently high precession angles their intensities tend to increase monotonically over a relatively wide thickness range, and the variations from reflection to reflection seen in the PED pattern remain relatively constant (Eggeman & Midgley, 2012*b* ▶). In other words, broadly speaking, strong reflections remain strong, weak reflections remain weak. This constancy leads to valid structure solutions, but comparison of individual intensities (as performed in conventional refinement) will lead to large *R* values.

The integration across the Bragg condition, which lies at the heart of the PED technique, has led some to use a two-beam dynamic correction (Blackman, 1936 ▶). This assumes that, for integrated diffracted intensities at small thickness, the behaviour is kinematic (intensities varying quadratically with structure factor and thickness), but at larger thicknesses the intensities vary linearly with structure factor and thickness. In general, multi-beam effects will limit the validity of this correction but it has been used with some success in certain cases (Klein, 2011 ▶; Hadermann *et al.*, 2012 ▶).

The large *R* values seen, even for apparently successful structure solutions, led the authors to propose a new method for refining PED diffraction data, namely rank refinement (Eggeman & Midgley, 2012*a* ▶), in which the model structure is refined so as to minimize not the absolute intensity difference across the reflections but the difference in rank, in other words to have an intensity ranking of the model structure (strong to weak) that best fits that of the experimental data.

**Figure 2:**
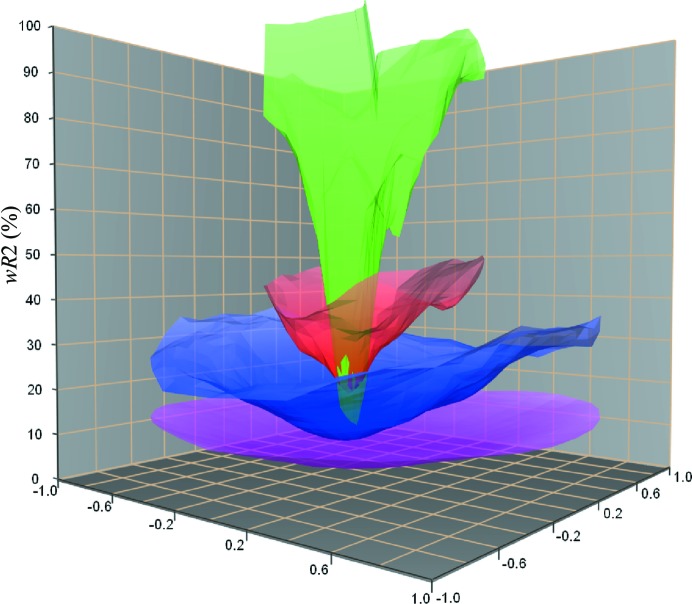
The weighted residual value *wR*_2_ as a function of tilt angle away from the ideal zone-axis orientation for Si 〈110〉 and as a function of precession angle from 0° (green) to 3° (mauve), indicating the reduced sensitivity of PED data to small misalignments. Reproduced from Palatinus *et al.* (2013 ▶).

Other error metrics are possible, for example minimizing the amount of negative (unphysical) potential in a real-space map, but perhaps the most robust method for refinement is simply to include the dynamic effects. PED data has recently been refined using dynamic diffraction theory (Palatinus *et al.*, 2013 ▶): in tests on known structures, the *R* factors dropped from 0.20–0.30 using a simple kinematic refinement to 0.05–0.08 using a full dynamic treatment. Work in the 1990s and later, using convergent beam electron diffraction (CBED) data to determine, for example, charge bonding density, was able to reach *R* factors of just a few per cent, but accurate values of orientation and thickness were essential for such high-quality refinements. Inaccurate refinement of thickness could lead to spurious results. With PED, it is clear that the pattern of intensities across the ensemble of reflections is, in general, far less sensitive to thickness, and an inaccurate orientation measurement (as shown in Fig. 2 ▶) may not have such a deleterious effect on atomic position refinement using PED data (Palatinus *et al.*, 2013 ▶).

The optimum precession angle has been studied by a number of groups and, as a rule of thumb, the precession angle should exceed (and ideally perhaps by a factor of two) the Bragg angle for the highest order reflection used in the structure solution. However, for practical reasons the chosen precession angle may be smaller than the ideal. For large angles, the aberrations of the probe-forming (objective) lens will increase the size of the probe. If parallel beams are used this is not a problem, but for convergent beams this must be taken into account. Early papers showed how, for a lens with spherical aberration (*C*_S_) dominant, the probe size varies linearly with convergence angle and quadratically with precession angle. In *C*_S_-corrected instruments, PED patterns have been recorded with nanometre-sized probes by balancing the diffraction limit (increasing with smaller convergence angle) and higher order aberrations (Eggeman *et al.*, 2013 ▶). The precession angle may also be limited by the geometry of the lattice and, particularly, by the need to avoid overlap between reflections in neighbouring Laue zones.

The apparent reduction in dynamic effects can be studied by considering homometric structures, which have the same structure factor amplitudes but different structure factor phases. By considering the diffraction pattern from two homometric structures, it is possible to gauge how dynamic effects are suppressed as the precession angle is increased (White *et al.*, 2010 ▶).

One factor that has been explored relatively little is the influence of inelastic scattering on the quality of the PED data. The integration of each reflection through the Bragg condition necessarily integrates not only elastic scattering but also inelastic and quasi-elastic (phonon-related) scattering. Both are especially problematic for thick specimens and when convergent beams are used, and can lead to an increase in the pattern background intensity. When measuring PED intensities, normally achieved by integrating across each recorded PED reflection (a Gaussian-like peak if a parallel beam is used, a disc if using a convergent beam), the background intensity under the peak must be removed by measurement of the local background next to each reflection (Kolb *et al.*, 2007 ▶). The presence of inelastic scattering in a diffraction pattern can be minimized by using an energy filter, either in-column or post-column, in which an energy-selecting slit is used to isolate the zero-loss peak and filter out the inelastic scattering above a certain energy loss (typically a few eV). The resulting pattern has much higher contrast, minimizing the background between reflections and the inelastic part of the Bragg peak intensity.

**Figure 3:**
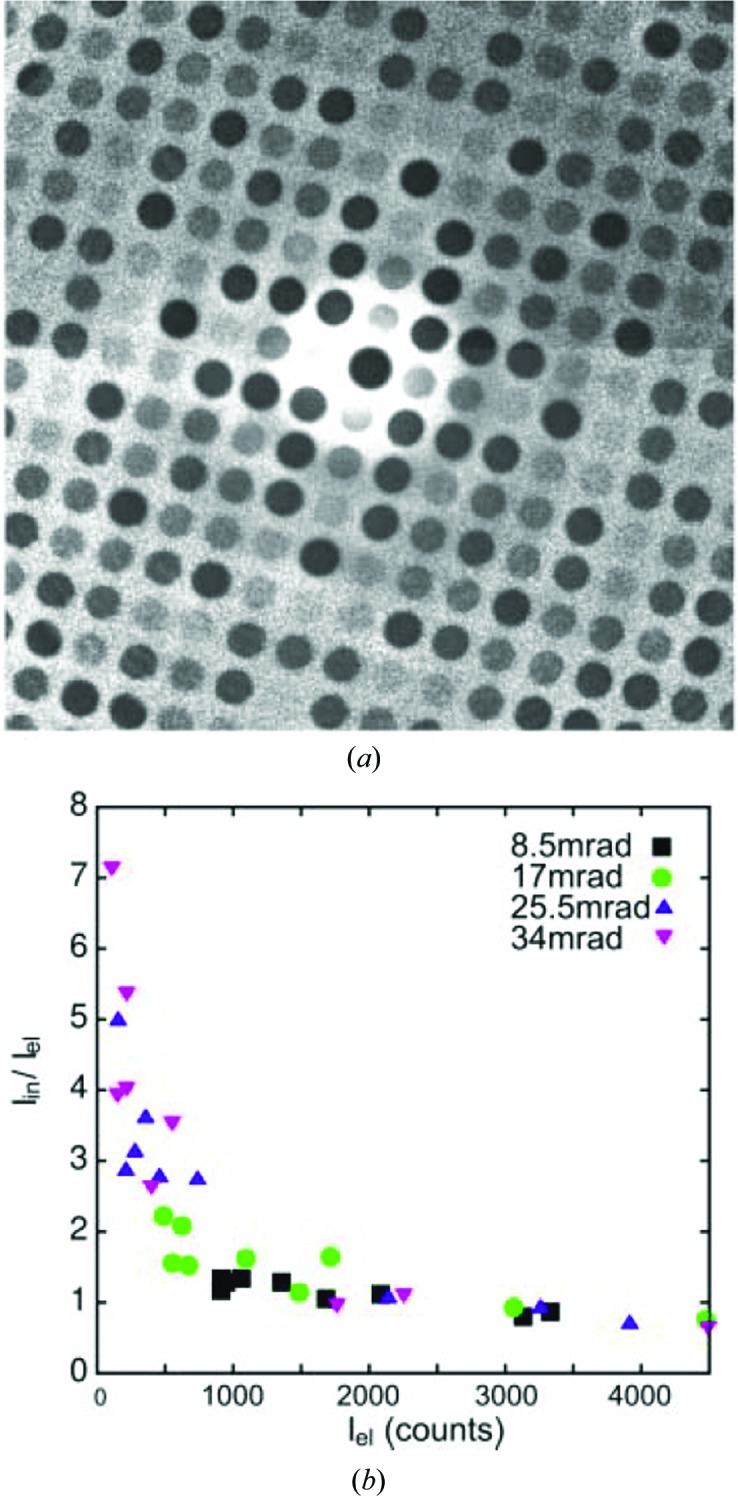
(*a*) The ratio of intensities from zero-loss energy-filtered and unfiltered PED patterns (with 34 mrad precession angle) for the [001] zone axis of Er_2_Ge_2_O_7_, indicating a significant variation in inelastic scattering in the diffraction pattern. (*b*) The calculated ratio of inelastic to elastic scattering as a function of elastic intensity. The increase at low intensity arises from the increase in the inelastic cross-section for lighter elements (contributing most to the weaker reflections). Reproduced from Eggeman *et al.* (2013 ▶), copyright 2013 Oldenbourg Wissenschaftsverlag GmbH.

Recent work by the authors (Eggeman *et al.*, 2013 ▶) showed how, by using energy-filtered PED, an improvement could be achieved in the *R* values following a simple kinematic refinement. A dynamic refinement would presumably produce similar improvements. Such elastic filtering is especially important for thick specimens, say 50 nm or more, where the majority of the scattering may be inelastic. If the pattern is not filtered, the inelastic contribution may be substantial and, in general, will not be distributed evenly across the reflections. The study measured the ratio of inelastic to elastic scattering in a PED pattern, presented in Fig. 3 ▶, and showed how this varied according to the relative contributions to the structure factor from heavy or light atoms, dominance of the former resulting in a much smaller ratio. This was attributed to changes in channelling from heavy to lighter atom columns, plus the variation in the total inelastic scattering cross-section, which varies as the inverse of the atomic number.

The geometry of PED data collection has also evolved in recent years. Improvements in computer control of the goniometer, and of the pre- and post-specimen scan coils, have led to developments in pattern collection. Software-based PED is now available where the beam precession is digital and, in principle, individual diffraction patterns may be collected at each angle around the azimuth, for integration *post facto* or for one-by-one analysis, the latter, with suitably fine digitization, allowing rocking curves to be recovered for each reflection (Zhang *et al.*, 2010 ▶).

In addition, rather than acquiring zone-axis PED patterns, for many systems, it is more profitable to acquire many patterns by tilting the sample about a single (initially unknown) crystallographic axis so as to build up a much fuller three-dimensional data set than is achievable through a few zone-axis patterns. This approach, known as ‘diffraction tomography’ or ‘automated diffraction tomography’ (ADT) (Kolb *et al.*, 2007 ▶), when combined with PED is fast becoming the technique of choice for *ab initio* structure determination using electrons. The ADT method will be discussed further in §3.2.

## 3. Recent applications   

Even though there is continuing debate about how and why PED works for structure solution given the inherent dynamic effects, and about the optimum experimental parameters, electron crystallographers have used PED to identify and solve many new phases. In this section we focus on some very recent examples; the brevity of this review does not allow us to be comprehensive but it does enable a snapshot to be given, illustrating the success of the method and diversity of the materials studied with PED.

### 3.1. Zone-axis patterns   

Traditionally, PED patterns, and indeed most electron diffraction patterns, have been recorded after the crystal of interest has been tilted to a major zone axis. This was primarily for three reasons. Firstly, the density of reflections (including HOLZ reflections) at such an axis is much higher, allowing a large data set to be collected in one acquisition. Secondly, the underlying symmetry of the crystal is revealed, allowing identification of the point and space group, especially if combined with conventional CBED patterns, highlighting in more detail the presence of Gjønnes–Moodie lines, for example. Thirdly, in using the data for structure solution, the low order (and possible high symmetry) of the axis would often lead to a reasonably complete view of the atoms, at least in projection. HOLZ data can provide atomic parameter information in the zone-axis direction. As such, zone-axis (ZA) patterns offer many advantages and are still used for structure solution.

**Figure 4:**
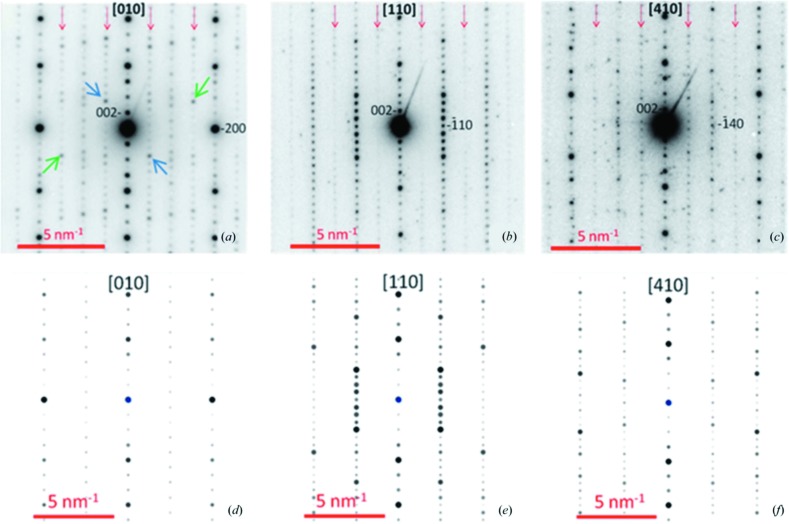
Experimental zone-axis PED patterns from Ce_10_W_22_O_81_ (top) compared with simulated patterns for the accepted cerium tungstate structure (bottom). Structural variations are indicated in the experimental patterns, including superlattice reflections (red arrows) and variations in point-group symmetry (blue and green arrows related by an inversion centre). Reproduced from Patout *et al.* (2014 ▶).

To illustrate the ZA-PED approach, we highlight some recent successes in solving structures or in garnering important crystallographic information. Mixed metal oxides are an important class of materials and are known to lead to a variety of structures modulated by small distortions of the metal–oxygen polyhedra. The exact nature of the distortions and consequent symmetry breaking can be difficult to determine with powder X-ray diffraction. The small probe available using PED can help enormously in this regard. Recent examples using ZA-PED include that of CaGd_2(1−*x*)_Eu_2*x*_(MoO_4_)_4(1−*y*)_(WO_4_)_4*y*_ (Morozov *et al.*, 2013 ▶), a scheelite-related compound that is a promising light-emitting material for photonic applications, including phosphor-converted light-emitting diodes (LEDs). Here the distortions, driven by cation vacancies, lead to the formation of an incommensurate superstructure with superspace group *I*2/*b*(αβ0)00. A similar study investigated the monoclinic superstructure found in ortho­rhombic Ce_10_W_22_O_81_ (Patout *et al.*, 2014 ▶). Here, ZA-PED patterns, in combination with high-resolution imaging and powder X-ray diffraction (XRD), were used to determine the space group and atom coordinates and to attribute the superstructure to a partial oxidation of Ce^3+^, leading to interstitial oxygen ions; evidence of this superstructure is seen in the PED patterns shown in Fig. 4 ▶. Quasicrystals and their approximants provide a severe challenge for structure solution. ZA-PED was used successfully to determine the structure of the approximant ∊_16_ of an Al–Rh structure (Li *et al.*, 2010 ▶) composed of over 150 unique atoms.

Structure solution using ZA-PED has also been applied to light beam-sensitive structures such as LiBH_4_, a material proposed for hydrogen battery storage. Sub-micrometre-sized crystals were examined in low dose conditions and high-quality structure solution and refinement resulted (Hadermann *et al.*, 2012 ▶). The correspondence between the experimental intensities and the kinematic intensities in the refined structure was improved in this case by application of a two-beam dynamic correction factor (Blackman, 1936 ▶), indicating that, at least for this crystal, multiple scattering is relatively limited and a (dynamic) two-beam approximation is reasonably valid.

The zone-axis geometry has also been applied to ordering within alloy structures and, in particular, to the measurement of the long-range order parameter, *S*, normally measured from the intensity ratio of a superlattice to a parent reflection. The fully ordered Fe–40%Al system was used as a test case and the intensity ratio for the 311 and 321 reflections was compared with simulations as a function of thickness (Ji *et al.*, 2010 ▶). With precession, the ratio changed monotonically with thickness (unlike the case without precession) and thus was shown to be well suited for order parameter measurements at the nanoscale. In a subsequent study, the ordering state in orthopyroxene (Jacob *et al.*, 2013 ▶) was investigated. Here, ZA-PED patterns were used to determine the Fe and Mg distributions on different octahedral sites in the orthopyroxene structure. In this case, the distribution can give clues as to the cooling history of the mineral, which is of interest in metamorphic rocks. Refinement of the structure was undertaken using a fully dynamic approach, and *R* factors less than 0.10 were achieved in the best cases.

### 3.2. ADT-PED   

An alternative to the zone-axis geometry, and one now preferred by many, is that of automated diffraction tomography (ADT) combined with PED (Kolb *et al.*, 2007 ▶). The ADT method is to acquire a tilt series of diffraction patterns about an initially unknown tilt axis, every degree or so, so as to sample reciprocal space in a systematic way and build up, slice-by-slice, a more complete three-dimensional view of the reciprocal lattice and the intensities of reflections at each reciprocal lattice point. The great advantage of this method is the lack of necessity to find major zone axes, saving time and reducing the likelihood of beam damage on beam-sensitive crystals. This is especially important for organic crystals, zeolites and microporous framework materials, where this technique has been of particular benefit in recent years (Kolb *et al.*, 2010 ▶, 2011 ▶). In addition, each pattern is likely to be away from a major zone-axis condition, minimizing the possibility of multiple scattering routes (although systematic and two-beam conditions may be strong at some, perhaps many, orientations within the tilt series).

PED provides an additional practical benefit in that the precessing motion integrates the signal across each reflection intercepted by the Ewald sphere, essentially ‘filling in the gaps’ between individual crystal tilts. This way, fewer reflections are likely to be missed, especially at higher scattering angles, resulting in a more complete data set. However, for slab geometries there will be a systematic change in the sample thickness – for example a factor of two at 60° tilt – which, if dynamic effects are important, may lead to erroneous intensities being used and difficulties in normalizing across the tilt series.

There are a number of variants on this diffraction tomography method, such as combining beam tilt and crystal rotation about a single tilt axis (‘rotation electron diffraction’ or RED; Willhammar *et al.*, 2014 ▶), and a digital precession method (Zhang *et al.*, 2010 ▶) in which patterns, at each crystal tilt, are recorded at multiple azimuthal angles and may be examined independently or summed to give a PED pattern. Here we focus only on the ADT-PED method and pick out some of the more recent highlights.

**Figure 5:**
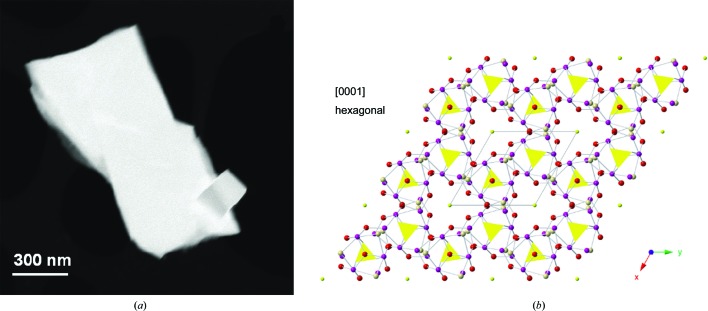
(*a*) STEM ADF image of a crystal of a previously unknown bismuth sulfate structure. (*b*) An [001] projection of the hexagonal structure recovered from the small crystal in the bottom right of part (*a*) using PED-ADT data. The atoms are colour-coded as follows: purple denotes Bi atoms, red O, pink OH groups and yellow (SO_4_)^2−^ and (S_2_)^2−^ anions. Adapted from Capitani *et al.* (2014 ▶) with permission from the Mineralogical Society of America.

**Figure 6:**
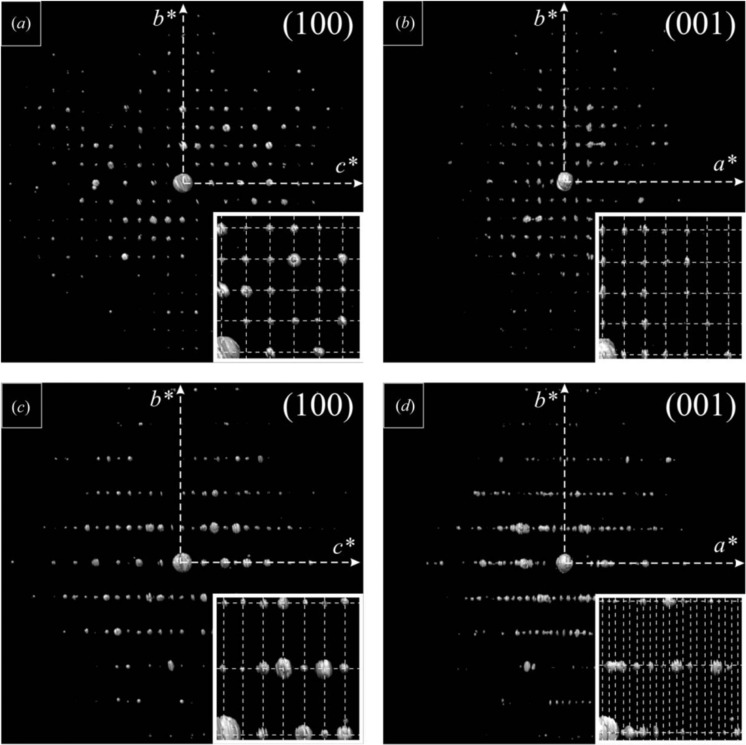
Projections of reconstructed three-dimensional reciprocal space at major zone axes recovered from PED-ADT analysis of oligo-*p*-benzamide structures. (*a*) and (*b*) are from OPBA3, while (*c*) and (*d*) are from OPBA4. Extinctions along the c* axis are visible in both (100) projections, as well as those due to the *c*-centring in OPBA4. The insets show magnified regions of the projections overlaid with the reciprocal lattice. Reproduced from Gorelik, van de Streek *et al.* (2012 ▶).

Since the introduction of the ADT-PED method (Kolb *et al.*, 2007 ▶), the development and more general use of modern structure-solution algorithms, including *SIR* (Burla *et al.*, 2007 ▶), *ENDEAVOUR* (Putz *et al.*, 1999 ▶), *SUPERFLIP* (Palatinus & Chapuis, 2007 ▶) and others, have allowed a wealth of structures to be investigated. In particular, the determination of organic and framework structures has benefitted from this method, including catalytically active bismuth–organic frameworks (Feyand *et al.*, 2012 ▶), complex zeolites (Smeets *et al.*, 2013 ▶; Jiang *et al.*, 2011 ▶), related sulfate structures (Capitani *et al.*, 2014 ▶; see Fig. 5 ▶), germano–silicate frameworks (Lorgouilloux *et al.*, 2013 ▶) and organic crystals (Gorelik, van de Streek *et al.*, 2012 ▶). The completeness of the diffraction data in this last example can be seen in Fig. 6 ▶, where major zone-axis projections of two different oligo-*p*-benzamide reciprocal lattices are shown. All the organic components were correctly recovered from the diffraction data.

The completeness of the electron diffraction data provided by ADT-PED also provides an opportunity to study complex inorganic crystal structures (Mugnaioli *et al.*, 2014 ▶; Pignatelli *et al.*, 2014 ▶) and cases where there is nanoscale distortion of the structure compared with the bulk (Gorelik, Sarfraz *et al.*, 2012 ▶), allowing variations in the mineralization of biological samples to be examined (Mugnaioli *et al.*, 2014 ▶). Incommensurate structures have also been studied with ADT-PED, with a recent structure solution found for an incommensurately modulated crystal of Bi_5_Nb_3_O_15_ (Boullay *et al.*, 2013 ▶).

### 3.3. Scanning PED   

By scanning the electron beam, as happens in the scanning electron microscope (SEM) or STEM, diffraction patterns can be acquired pixel-by-pixel to build up a four-dimensional data set in which two-dimensional reciprocal (crystallographic) information is available at each two-dimensional real-space position. This type of scanning diffraction technique has been used for some years to identify domain structures and changes in orientation across the field of view. Recently, however, a scanning variant of PED (SPED) has been developed in which PED patterns are acquired at each pixel (Rauch *et al.*, 2008 ▶). The advantages of PED, as exemplified for structure determination, remain namely that a far greater number of reflections are seen in a PED pattern than in a conventional (static) diffraction pattern, and the intensities are in some sense ‘more kinematic’ in nature. SPED has been developed primarily as a technique complementary to electron back-scatter diffraction (EBSD) (Schwartz *et al.*, 2009 ▶) in an SEM, to provide orientation information in the form of overall grain structure, texture, Euler maps, inverse pole figures, misorientation *etc*.

Technically, the addition of a scan to PED is relatively straightforward. An additional signal is given to the pre-specimen deflectors to control the scan size and increment, but the scan speed must be synchronized with the precession speed so as to ensure a full azimuthal scan at each pixel. As with EBSD, the recorded PED pattern is compared with a library of possible structures or patterns in an automated fashion, leading to very rapid identification. The requirement for high spatial resolution and well sampled data leads to reduced dwell times (to ensure a reasonable total acquisition time) and thus, with current commercial detectors and cameras, each individual PED pattern has a relatively low signal-to-noise ratio (SNR). With improved camera configurations in the future, using highly sensitive and efficient direct electron detectors, high SNR PED patterns may be obtained with even a relatively low dose. To achieve reliable pattern matching, the more reflections seen in the acquired pattern the better, and thus a large precession angle is desirable. However, this then limits the angular resolution achievable in the determination of the sample normal, and so in practice a compromise is struck and a typical precession angle chosen is 0.5°.

**Figure 7:**
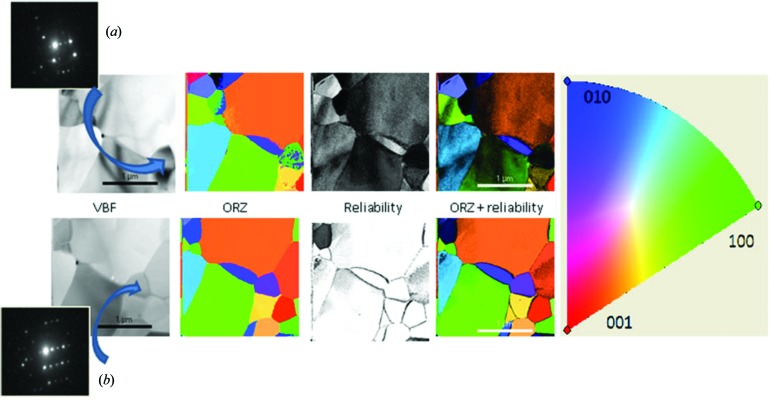
Scanning electron diffraction analysis of polycrystalline alumina, showing maps of virtual bright field (VBF) intensity, orientation (according to the included colour-scheme) and reliability of indexing for both (*a*) conventional and (*b*) precession electron diffraction. Reproduced from Viladot *et al.* (2013 ▶) with permission from John Wiley and Sons on behalf of the Royal Microscopical Society.

Fig. 7 ▶ shows a recent example of SPED in which the grain orientation of polycrystalline alumina is examined. For each pattern a reliability index is obtained, indicating the difference between the first and second highest pattern matches for the indexing, and thus the likelihood that the phase and orientation have been correctly and uniquely identified. The figure shows how SPED provides a better quality map than if conventional patterns are used. A virtual bright-field (VBF) or dark-field (VDF) image is possible simply by outputting the intensity per pixel associated with a point (or area) in reciprocal space – typically, but not necessarily, a Bragg reflection.

In addition to providing a larger number of (ZOLZ and HOLZ) reflections to aid accurate indexing, PED also improves the fidelity of the results (Rauch *et al.*, 2010 ▶; Moeck *et al.*, 2011 ▶) by effectively smoothing out small variations in orientation (through the rocking action of the precession), thus aiding phase identification. A reduction in dynamic effects on the diffraction pattern further improves the comparison of intensities between experimental data and (kinematic) simulation, increasing the fidelity of any pattern matching and improving the reliability index. Finally, PED helps to reduce the structured background visible in many diffraction patterns, arising typically from Kikuchi diffraction, by averaging across a large number of orientations and thus increasing the reliability of peak-finding algorithms (Estradé *et al.*, 2012 ▶; Viladot *et al.*, 2013 ▶).

The use of SPED, in essence as a TEM analogue of EBSD, has gathered momentum quickly and there are now many examples of its use. Early work focused on relatively simple face-centred cubic (f.c.c.) metal systems such as polycrystalline aluminium, palladium or copper (Moeck *et al.*, 2009 ▶; Rauch *et al.*, 2010 ▶), but it has now been extended to more complex problems such as twinning in hexagonal alloys (Zhang *et al.*, 2013 ▶). The high spatial resolution achievable has allowed extensive research into nanoscale samples, from iron oxide (Rouvimov *et al.*, 2009 ▶) through to metallic multilayer stacks (Liu *et al.*, 2011 ▶).

**Figure 8:**
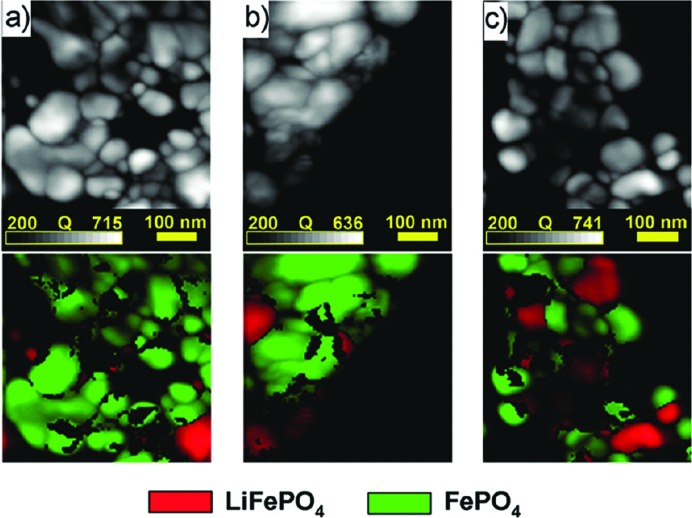
Quality-index (*Q*) maps and false-colour phase maps for lithium-inserted iron phosphate samples with (*a*) 89%, (*b*) 80% and (*c*) 50% charging of lithium. This indicates that each particle fully charges/discharges its lithium content and there are no particles with a mixed structure of both LiFePO_4_ (red) and FePO_4_ (green). Reprinted from Brunetti *et al.* (2011 ▶), copyright 2011 American Chemical Society.

The newer developments in this area utilize the wealth of diffraction data recorded in the experiments and the flexibility of the TEM for performing *in situ* experiments, for example studies of phase change during oxidation and reduction (based on studies of Ni-YSZ electrodes; Jeangros *et al.*, 2010 ▶). Increasingly, however, it is the ability to utilize the sensitivity of the diffraction data post-acquisition that offers some of the greatest opportunities for this technique. Already studies have been performed on sub-grain boundaries in highly defective structures (Cizek *et al.*, 2012 ▶), the crystalline variations (or lack thereof) in nanoparticles with differing morphologies (Voliani *et al.*, 2014 ▶) and the possible mechanisms for lithium migration in battery materials (Brunetti *et al.*, 2011 ▶). This last example is shown in Fig. 8 ▶, where the lithiated (green) and unlithiated (red) variants of iron phosphate are mapped. This, coupled with the power of forming VDF images to study crystallographic features such as dislocations (Rauch *et al.*, 2014 ▶), suggests that the use of scanning diffraction analysis, and SPED in particular, is set to become a powerful and widely used technique for materials characterization.

The sensitivity of PED to changes in local crystal orientation has recently been pushed further to study strain in semiconductor devices. In some semiconductor structures, strain may be introduced deliberately to alter the local electronic behaviour, and there is a pressing demand from the semiconductor industry for techniques to map such strain at the nanometre level. Rouviere and co-workers (Rouviere *et al.*, 2013 ▶) have shown how, by using a small PED beam, it is possible to map strain in a transistor structure with a precision of *ca* 10^−4^ using a probe of *ca* 3 nm in size.

## 4. Complementary techniques   

PED (and its scanning variant SPED) offers a broadly applicable technique that enables the structure and orientation of the underlying crystal to be studied rapidly and with reliability. However, one must not rule out additional, complementary, techniques that, in some circumstances, can offer improved analysis (sensitivity to symmetry determination, strain, lattice parameter determination *etc*.). Foremost amongst the alternatives for structure determination is high-resolution imaging (Hovmöller *et al.*, 2002 ▶), which can be particularly beneficial for the study of thin weakly scattering crystals [zeolites, mespororous materials, metal–organic frameworks (MOFs) *etc.*], where dynamic effects are minimized and the phase information encoded in the crystalline image contrast can be used to determine structure factor phases, or at least phases associated with the Fourier transform of the exit wavefunction. In some cases, a combination of PED and imaging, in addition to powder X-ray diffraction, offers an optimal combined solution to overcome possible limitations with each individual technique (*e.g.* a lack of completeness in PED data, overlapping peaks in XRD) (Xie *et al.*, 2008 ▶; McCusker & Baerlocher, 2013 ▶). In the future, a consistent structure that satisfies results from a combination of techniques may be the most reliable way forward.

For the determination of symmetry or very small local strains, CBED can provide the most accurate way to determine these from diffraction data. If the crystal quality is good, high-order deficiency lines in the zero-order disc (or sometimes excess lines in the HOLZ) of CBED, or indeed large-angle CBED (LACBED), patterns can be used to determine very subtle symmetry breaking or changes in lattice parameter (strain). A scanning variant of CBED can also be used to determine, for example, spatially varying symmetry breaking in silicon (Kim & Zuo, 2013 ▶) and ferroelectric Pb(Mg_1/3_Nb_2/3_)O_3_–31%PbTiO_3_ crystals (Kim *et al.*, 2013 ▶).

The vast improvement in computer control of microscopes now allows many of these diffraction techniques to be acquired in a ‘digital’ format. Specifically, there are two similar methods [digital, or D-, LACBED (Beanland *et al.*, 2013 ▶) and large-angle rocking-beam electron diffraction, or LARBED (Koch, 2011 ▶)] for acquiring LACBED patterns using a focused beam. Previously, the most popular method for LACBED acquisition was that devised by Tanaka *et al.* (1980 ▶), in which the sample is raised above the image plane so as to allow the selected-area (SA) aperture to select a single beam and enable a single LACBED disc to be viewed on the screen. Modern methods allow the acquisition of multiple beams from a focused (*ca* 10–20 nm) spot by rocking the beam (over perhaps a few hundred milliradians) in a serial way, akin to the rocking-beam mode in STEMs and SEMs. The vastly expanded range of reciprocal space seen within LACBED patterns allows a greater certainty in the determination of symmetry and, with suitable energy filtering, may provide an improved route for dynamic structure refinement.

## 5. Conclusions   

The adoption of PED as the electron crystallographic technique of choice to determine unknown structures or local orientation changes has increased partly through the advent of modern commercial third-party hardware, partly through the greater ability to control the microscope easily through software (scripting) or hardware (*via* relatively straightforward connection to scan coils), and partly through the increase in computational power that allows fast processing and analysis. *Ab initio* structure solution of inorganic and small organic crystal structures is becoming routine, with the combination of PED and diffraction tomography perhaps providing the ideal combination. The recent development of direct electron detectors has provided a huge impetus to the study, using PED, of highly beam-sensitive samples, including silicates and metal–organic structures, and even protein crystals (Nicolopouos, 2014 ▶). Residual dynamic effects may still cause problems though, manifesting either through increased dynamic interactions at, or near, zone axes and strong systematic rows, or by changes in the probability of multiple scattering with an increase in thickness as the sample is tilted.

Whilst structure solution of unknown structures with PED has proven to be highly successful, structure refinement, assuming the intensities are kinematic, consistently leads to high *R* factors, typically 0.20–0.30. The likely major reason for this, in the vast majority of cases, derives from the simple fact that individual PED intensities, indeed all electron diffraction intensities in general, are not kinematic. The reason structure solution is possible is that the ensemble of PED intensities behaves in a kinematic-like way. Put simply, as the sample thickness increases, on the whole strong reflections remain strong and weak reflections remain weak. This ‘pattern’ of intensities does not change too much with thickness, as long as the precession angle is sufficiently large, and as a rule of thumb this should be greater than the Bragg angle of each reflection used. New ways to refine the structure using PED intensities must be found. Rank refinement is one but perhaps dynamic refinement may ultimately be the best way forward. However, for that to be successful a thickness determination is needed (or must be measured independently, perhaps using CBED or EELS) and energy filtering would be advantageous, especially if considering relatively thick specimens (>1/3 total inelastic mean free path). Nevertheless, it is remarkable how PED has contributed so successfully to the development of electron crystallography and the determination of atomic structure.

The development of SPED has increased rapidly since the introduction of fast pattern acquisition and fast template matching. It will be interesting to see how far the sensitivity of the technique can be pushed, but its use to determine strain in semiconductor structures is already an exciting advance. Mapping orientation in three-dimensions using SPED has recently been realised (Eggeman *et al.*, 2014 ▶) and orientation relationships considering sub-volumes of material are now a possibility, providing a complementary higher-resolution technique to the three-dimensional diffraction tomography methods available with X-ray synchrotron imaging (Ludwig *et al.*, 2008 ▶; Bleuet *et al.*, 2008 ▶) and an alternative, more electron-efficient, method to existing TEM-based methods (Liu *et al.*, 2011 ▶).
